# Creatine Monohydrate as an Effective Supplement for Muscular Fatigue in an Ehlers-Danlos Patient

**DOI:** 10.7759/cureus.61721

**Published:** 2024-06-05

**Authors:** Christopher R Behringer, Shezda Afrin

**Affiliations:** 1 Clinical Education, Lake Erie College of Osteopathic Medicine, Erie, USA; 2 Family Medicine, T.J. Samson Community Hospital, Glasgow, USA; 3 Arts and Sciences, University of Pennsylvania, Philadelphia, USA

**Keywords:** connective tissue disorder, myalgia, muscular endurance, creatine monohydrate, chronic muscular weakness, ehlers danlos syndrome (eds)

## Abstract

Patients with Ehlers-Danlos syndrome (EDS) frequently report symptoms such as chronic pain and muscular fatigue that can heavily impact their quality of life. The treatment for many of the physical symptoms of EDS is focused on supportive care, which may include physical therapy and exercise programs. However, many patients will experience difficulty in deriving benefits from these activities due to significant pain and fatigue from physical activity. We report a case of a 39-year-old female with a history of EDS whose physical capabilities were severely impacted by their chronic pain and fatigue symptoms. After little progress was made with their current treatment plan of analgesics, manual therapy, exercise, and physical therapy, the patient was supplemented with creatine monohydrate due to its studied benefits in muscular strength and endurance for athletes. Following supplementation, the patient reported significant benefits in their muscular fatigue symptoms, allowing them to engage in daily activities and exercises more effectively. This case demonstrates a potential addition to the treatment of EDS that can improve a patient's quality of life.

## Introduction

Ehlers-Danlos syndrome (EDS) is a group of connective tissue diseases with 13 subtypes recognized under the 2017 EDS Classification System and affects one’s collagen production, especially in the extracellular matrix [1]. Patients commonly suffer symptoms of muscle weakness, myalgias, and easy fatigability. The syndrome primarily affects the joints and skin [2] and results in osteoarticular pain [3]. Chronic joint and limb pain, along with tissue bruising and skin tearing, are common symptoms, amongst various others [4]. There are also reported abnormal collagen fibrils. These fibers are essential for tissue stability [5]. The treatment for most physical symptoms of EDS is supportive in nature, such as physical therapy and exercise programs. However, many patients suffer from extreme discomfort in even daily activities, and they may suffer from demotivation and an inability to progress effectively in their physical rehabilitation programs. The reasons for this inability can be from symptoms such as pain or muscular fatigue. This can lead to a cycle of pain, decreased movement, and deconditioning, which can lead to worsening fatigue and pain. Other treatments, though short term, are anti-inflammatories and corticosteroids. Long-term use of such medications is not recommended, especially in cases of EDS. For example, long-term systemic corticosteroids are not recommended in patients with skin fragility, which can occur in certain subtypes of EDS, as they can increase skin thinning and fragility and actually inhibit collagen synthesis [6]. This can leave many patients and clinicians feeling significantly challenged in improving the quality of life in EDS patients. Creatine monohydrate, 95% of which is stored in the muscles [7], has been well-studied in athletes and strongly suggests benefits such as increases in muscular strength and endurance, especially in short bursts of high-intensity exercise. However, its use in aerobic endurance is still debated [2,7,8]. Creatine monohydrate may provide similar improved muscular endurance and strength in EDS patients as they engage in daily activity and physical therapy as these do not usually incorporate an aerobic component. A literature review on PubMed reveals no reports on the usage of creatine monohydrate in the treatment of chronic muscular weakness and fatigue. We hope that this may serve as a new treatment for patients suffering from muscular fatigue in EDS patients and improve their quality of life.

## Case presentation

**Figure 1:**
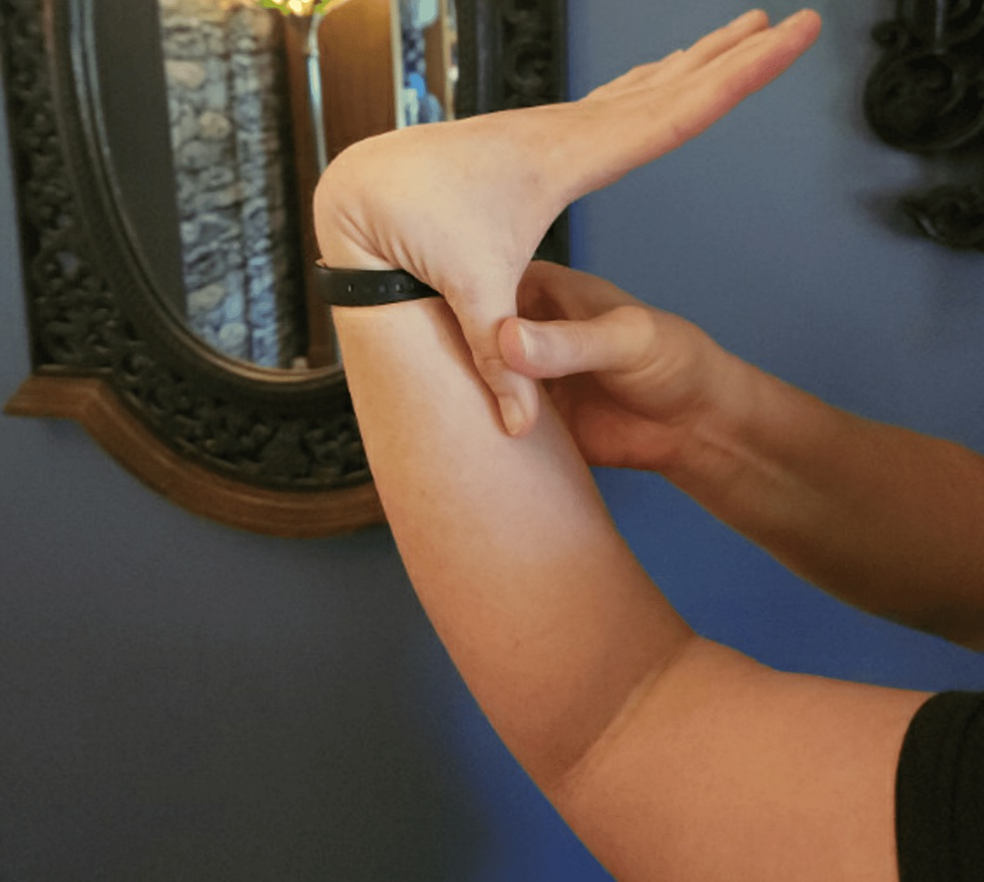
Example of the patient's hypermobility

A 39-year-old female patient, with a past medical history of hypermobile EDS (Figure 1), dysautonomia, obesity, and postural orthostatic tachycardia syndrome, sought care for her bilateral hip and shoulder pain secondary to her EDS in 2023. Prior to her formal diagnosis of EDS, the patient had symptoms of chronic right hip and shoulder pain. She initially responded well to physical therapy and dry needling. However, in 2019, the patient began having left-sided hip and shoulder pain without inciting injury or insult. Her primary care provider at the time was concerned about an auto-immune process and sent the patient to rheumatology. After extensive workup, she was deemed to have EDS in 2020 with hypermobile EDS being confirmed after seeing a geneticist. She began treatment with continued physical therapy, home exercise programs, and pharmacotherapy for pain management with minimal improvement. In December 2023, the patient presented to a new clinic for re-evaluation and consideration of treatments, such as osteopathic manipulation and acupuncture. Initial treatment with osteopathic manipulation and acupuncture yielded transiently beneficial results. She said that these interventions helped in the short term but her symptoms returned, limiting her ability to pursue activity. After several weeks, the patient mentioned that one symptom that was particularly severe was a sensation of muscular fatigue, limiting her ability to engage in activity separate from her pain. It was then recommended for the patient to try supplementation with creatine monohydrate due to its well-established benefits in muscular strength and endurance in athletes [2]. A loading phase of 20 g daily for one week, followed by a maintenance dose of 3-5 g daily, was recommended as this protocol is adequate to maintain muscular concentrations of creatine [8]. After one month, she reported a significant benefit in her muscle weakness, myalgias, and even overall level of pain within her current activity levels. At this time, the patient was now able to complete desired household chores, such as cooking and cleaning the dishes in a single effort. Additionally, she was able to engage in resistance training at the local gym. They reported no gastrointestinal (GI) upset after starting supplementation. As a result, the patient desired to try coming off of her anti-inflammatory (diclofenac 50 mg twice per day (BID)). One month after cessation of the anti-inflammatory, and two months after beginning supplementation, she reported that her muscular fatigue had continued to subside. The patient reported that prior to supplementation they could walk 10-15 minutes and required a 10-15 minute rest afterwards. However, after supplementation, she could now walk 20-25 minutes with a 10-15 minute rest. Another activity she reported that she could now do was washing and brushing her hair in a single effort without significant pain or fatigue in her shoulder girdle. However, she started experiencing a return of her previous pain levels beyond these activity levels. She desired the re-initiation of the anti-inflammatory, and it was restarted. Of note, the patient would differentiate the difference between her pain, general fatigue, and muscular fatigue. In terms of the muscular fatigue symptoms, they would describe it as not being in pain, and not being tired, but feeling as if they were "walking thru water, like in a swimming pool". It was this sensation, in particular, that the patient reported benefit in post supplementation.

## Discussion

Therapeutic protocols, corticosteroids, and analgesics (opioids, nonsteroidal anti-inflammatory drugs (NSAIDs), etc.) are not always effective in improving the quality of life among EDS patients [3]. This case describes a patient with EDS who received a noticeable improvement in her muscular fatigue after supplementation with creatine monohydrate. To the authors’ knowledge, this is the first report of its kind in the medical literature. After performing a loading phase and transitioning to a maintenance phase, the patient experienced an improvement in her symptoms over time. The patient could now complete activities, such as cooking and cleaning, without excessive fatigue. She could also engage in exercise programs at her local gym without excessive pain or discomfort while still on anti-inflammatory medication. Removal of the anti-inflammatory medication resulted in the return of her pain, but there was still improvement in her muscular fatigue symptoms and no reported regression in her ability to complete tasks. There are multiple benefits to taking creatine as a supplement. Not only is it an antioxidant and helps lower inflammation in the human body, but also it improves post-exercise recovery [7-9]. This helps explain why individuals may report lower levels of pain even after doing physical movements. Nutritional creatine is found in red meat and seafood, but commercial supplements have a greater level of the compound. Our body makes this amino acid via the pancreas, kidneys, and liver, and it can be found mainly in our muscles but also in the brain [10]. The supplement has also shown benefits in enhancing bone strength, which can aid in preventing falls for older patients [10,11]. A study in the Netherlands investigated the positive correlation between muscle weakness and fatigue severity in EDS patients [12]. This can be due to the myopathy and/or polyneuropathy caused by EDS, which then results in a lack of physical activity. Without much movement, particularly due to the constant pain, deconditioning and worsening fatigue are difficult to avoid. In addition to many other health benefits, such as aiding in post-viral fatigue syndrome, one week of creatine supplementation amongst healthy adults increased skin capillary density, which is essential for exercise tolerance due to improved tissue oxygenation [13]. This helps alleviate physical fatigue after exertion. One significant benefit of creatine as a supplement is it poses no known severe adverse side effects in otherwise healthy individuals [13,14].

**Figure 2:**
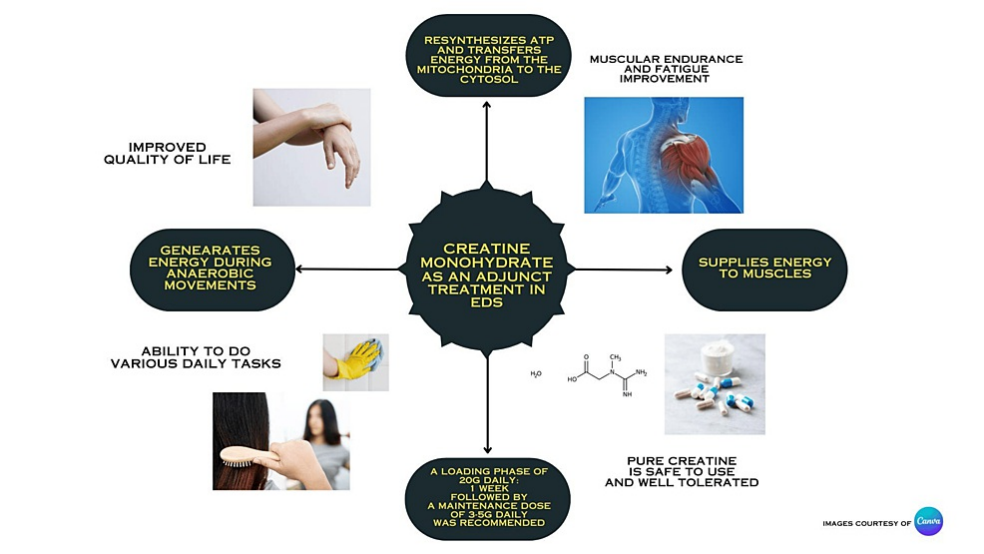
Summary of the use and benefits of creatine monohydrate The figure was designed by Shezda Afrin. Images courtesy of Canva under Creative Commons License.

In regard to safety, creatine monohydrate overall has a well-established safety profile as long as purity can be assured [9]. Unlike many supplements, it is relatively easy to find creatine monohydrate that has passed high-quality third-party testing. As it is commonly used in professional athletics, many brands will go through extensive testing to be considered World Anti-Doping Association compliant, and suppliers will publish their third-party testing results for purity. Concerns about creatine monohydrate causing renal damage are not consistent with the data [9]. Caution should be used in patients with existing renal disease, particularly being mindful of the dosage and quality of the supplement [14]. However, due to creatine monohydrate’s overall well-tolerated nature and established benefits in athletic patients, it may serve as an inexpensive and effective way of improving the livelihood of patients suffering from chronic muscle fatigue, secondary to EDS [13,15]. Due to this patient’s improvement, further research involving more patients may be warranted, as well as using a measurement tool to quantify the patient’s improvement. Due to creatine’s general improvement in muscular endurance and fatigue, further research may also be warranted in patients suffering similar symptoms from other conditions such as connective tissue disease and fibromyalgia. The use and benefits derived from creatine monohydrate by this patient are summarized in Figure 2.

The limitations of this report include that the total study time for this patient at the time of authorship is two months in a single patient. This is a relatively short period of time for most clinical studies, and a longer research period with more patients is warranted. However, the decision for such a short follow-up period was deliberate as a one-week loading phase of creatine of 20 g for one week, followed by a maintenance dose of 5 g daily, is a well-established protocol that is effective as increasing creatine stores in muscle cells [8]. The goal was to highlight the potential benefit of creatine as a relatively fast-acting agent in the improvement of symptoms for patients with EDS. Another limitation is the lack of standardized measurements of strength and endurance pre and post supplementation. It is the hope of the authors that this case report inspires further research that would include these measures.

Patient's Perspective

"Just one month on creatine helped me feel closer to my old, healthy self, more than any other previously prescribed medications/treatments."

"My previous treatments (PT, muscle relaxers, etc.) offered little-to-no benefit to my quality of life. Simple tasks such as grooming, basic housework, and walking were not positively affected in any way. I understand physical conditioning is a primary treatment for EDS; however, my overall pain and fatigue were not alleviated through treatments/meds effectively enough to begin targeted strength training. Since creatine, my pain has been mildly reduced, and the most notable benefit has been an overall decrease in muscle fatigue. This has allowed me to begin treating my condition more effectively. Furthermore, my reduction in fatigue has allowed me to function at a high enough level that I feel able to seek part-time employment for the first time in four years."

"Going forward, it is my sincere hope that others with EDS whose lives are heavily impacted, such as mine, can find relief and see a path to functional freedom. For the first time in four years, I have begun to have hope for a healthy and active life. I believe everyone affected by EDS should try this creatine regimen, so they, too, can reclaim their lives."

## Conclusions

Patients with EDS frequently experience symptoms of pain, fatigue, and muscular weakness. These symptoms can significantly impact the quality of life and activities of daily living. We reported a case where creatine monohydrate, of good quality, was used as an adjunct treatment for the patient due to its well-established benefit in muscular strength and overall athletic performance. After multiple treatment options were tried with no major improvement, it was recommended for the patient to incorporate creatine supplementation to alleviate her symptoms. The patient reported benefits in her ability to complete daily activities and engage in exercise programs more effectively. This case highlights the use of creatine monohydrate as an adjunct treatment in EDS patients suffering from symptoms of muscular weakness and fatigue.
